# Acute Pancreatitis Complicated with Choledochal Duct Rupture

**DOI:** 10.1155/2011/413268

**Published:** 2011-10-03

**Authors:** M. Bouvry, K. Van Renterghem, A. Verrijckt, P. Smeets, V. Meersschaut, S. Vande Velde, R. De Bruyne, M. De Vos, M. Van Winckel, S. Van Biervliet

**Affiliations:** ^1^Paediatric Gastro-Enterology, Ghent University Hospital, De pintelaan 185, 9000 Ghent, Belgium; ^2^Paediatric Surgery, Ghent University Hospital, De pintelaan 185, 9000 Ghent, Belgium; ^3^Paediatric Intensive Care, Ghent University Hospital, De pintelaan 185, 9000 Ghent, Belgium; ^4^Paediatric Radiology, Ghent University Hospital, De pintelaan 185, 9000 Ghent, Belgium; ^5^Adult Gastro-Enterology, Ghent University Hospital, De pintelaan 185, 9000 Ghent, Belgium

## Abstract

Recurrent acute pancreatitis is a rare clinical entity in childhood with unknown incidence (Rosendahl et al., 2007) and often occurring in a familial context. Genetic factors such as PRSS1 mutations (cationic trypsinogen gene) can be found in some patients. However, many remain idiopathic. The natural history remains poorly documented and the most frequent complications reported are pain, exocrine pancreatic insufficiency, diabetes mellitus, and pancreatic adenocarcinoma after long-standing hereditary pancreatitis. We describe a patient with hereditary pancreatitis in whom a mild pancreatitis episode was complicated by a perforation of the ductus choledochus.

## 1. Introduction

Recurrent acute pancreatitis is a rare clinical entity in childhood with unknown incidence [1] and often occurring in a familial context. Genetic factors such as PRSS1 mutations (cationic trypsinogen gene) can be found in some patients. However, many remain idiopathic. 

The natural history remains poorly documented and the most frequent complications reported are pain, exocrine pancreatic insufficiency, diabetes mellitus, and pancreatic adenocarcinoma after long-standing hereditary pancreatitis.

We describe a patient with hereditary pancreatitis in whom a mild pancreatitis episode was complicated by a perforation of the ductus choledochus.

## 2. Clinical Case

A 13-year-old girl, with recurrent pancreatitis; was transferred due to clinical deterioration 3 days after the start of an episode of acute pancreatitis. The familial history revealed a recurrent pancreatitis in her brother and in a maternal aunt and 2 nieces. The aunt and nieces had a proven PRSS1 mutation: p.N29I. The patient herself had four documented episodes of acute pancreatitis since the age of 18 months. It was presumed that she also had a hereditary pancreatitis, but she was never genetically tested. There were normal ultrasound studies of the biliary tree in the past.

She presented with abdominal pain, low-grade fever (38°C) and severe right shoulder pain.

Although hemodynamically stable with normal lung and cardiac auscultation, she presented with Cullen's sign. The right iliac fossa was tender with resistance. 

The laboratory findings showed leukocytosis 16.31 × 10^3^/*μ*L (normal (nl.) 4.5–12), C-reactive protein 25.1 mg/dL (nl. 0–0.5), hyponatremia (133 mmol/L (nl. 135–144)), low albumin (3.0 g/dL (nl. 3.4–4.8)), only slightly increased amylase 125 U/L (nl. 0–100), and lipase 61 U/L (nl. 0–41). The liver enzymes were normal, but bilirubin (1.8 mg/dL (nl. 0.3–1.2)) and *γ*-glutamyl transferase (52 U/L (nl. 4–22)) were slightly increased. 

**Figure 1:**
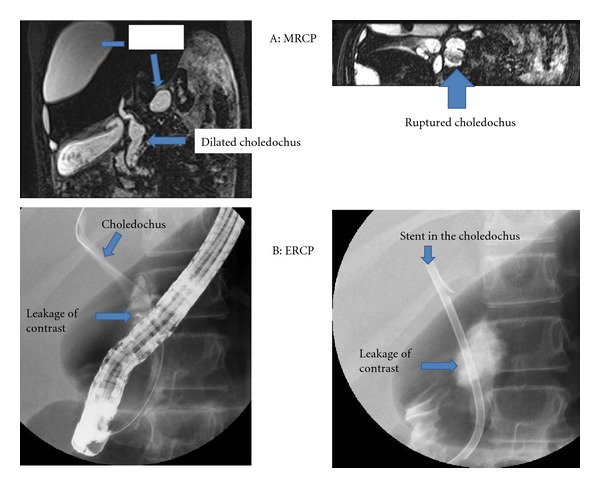
A: MRCP images demonstrating the subcapsular effusion, dilatated choledochus, and the choledochus rupture. B: ERCP images from leaking choledochus and stent placement.

The magnetic resonance cholecystopancreaticography (MRCP) showed a fluid collection lateral to the liver, probably subcapsular. A fusiform dilatation of the common bile duct (CBD), with a posterior interruption of the CBD and a fluid collection behind the duct, was visualised (Figure 1). She was treated with intravenous antibiotics and parenteral nutrition due to enteral food intolerance.

Punction of the perihepatic fluid collection resulted in 700 cc of intense bile stained fluid with concentration of amylase (2140 U/L) and lipase (2220 U/L). A streptococcus viridans was cultured from this fluid. 

The endoscopic retrograde cholecystopancreaticography (ERCP) confirmed dilatation of the CBD with periductal leakage of the contrast fluid (Figure 1). There was no stone detected. After endoscopic sphincterotomy, a stent was placed across the lesion. After drainage of 600 mL reactive ascites (without bile staining or amylase activity) and another 700 mL of perihepatic fluid, she improved and was discharged after 17 days.

**Figure 2:**
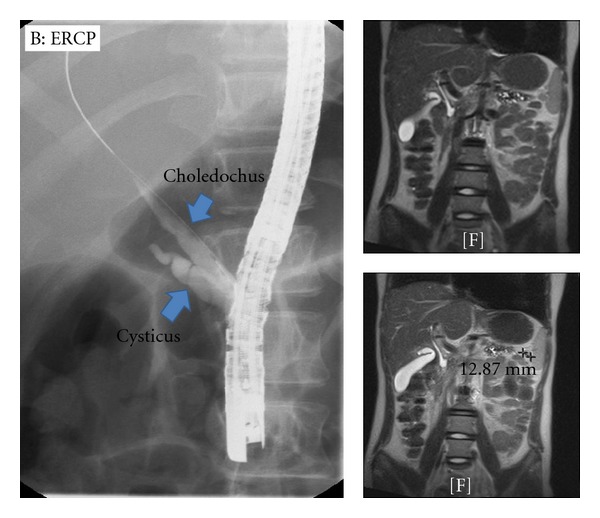
A: MRCP images demonstrating normalisation of the biliary tree after removal of the stent. B: ERCP image from choledochus after stent removal.

Two months later, the choledochal stent was removed and the cholangiography showed a normal biliary tree. The cystic duct has a broad opening towards the choledochal duct (Figure 2). The MRCP confirmed a normal intra- and extrahepatic biliary tree. It was decided to abstain from further interventions based on these images. A PRSS1 mutation: p.N29I was also confirmed in this patient.

Due to recurring pancreatitis booths, a second sphincterotomy with stenting of the Wirsung duct was performed 6 months after the initial procedure. During this procedure, a protein plug was removed from the Wirsung duct.

## 3. Discussion

This girl has a classical dominant mutation for hereditary pancreatitis in the PRSS1 gene: p.N29I [2]. Although there is a variable penetrance, patients frequently develop their first symptoms of pancreatitis around the age of 10 years, with an earlier onset when the inheritance is maternal [2]. This girl, inheriting her p.N29I from her mother, has had acute pancreatitis episodes since the age of 1.5 years. 

The development of pancreatic duct lesions and calcifications is frequent complications in patients with hereditary pancreatitis [2]. About 1/3 develops exocrine and/or endocrine pancreatic insufficiency [2]. Up to now there is, however, no case described of choledochus perforation in the context of a pancreatitis episode. 

Due to the frequent association of pancreatitis with choledochal cysts and/or long common channel, high resolution ultrasound and MRCP are necessary to exclude choledochal cysts in children with recurrent pancreatitis [3, 4]. In this girl, however, due to the familial context, only an ultrasound was performed prior to the last complicated pancreatitis episode.

Bile duct perforation with biliary ascites or retroperitoneal bile effusion is a rare clinical entity in childhood [5]. Although there are articles describing spontaneous biliary ascites [6], several authors have doubts about the existence of this entity [5]. Most of the described cases are associated with choledochal cysts or an abnormal pancreaticobiliary junction [5, 7–9]. 

Recurrent pancreatitis has been described as a contributing factor in the formation of choledochal cysts [10]. Ng et al. describe three cases with a documented normal CBD who developed a choledochal cyst secondary to several episodes of pancreatitis [11]. This, however, cannot be the cause of the perforation in this girl since the extra- and intrahepatic bile ducts were normal after removal of the stent. 

There are no arguments for cholelithiasis on any of the imaging techniques used. But since the problem resolved after endoscopic sphincterotomy and stenting, stenosis or obstruction by protein plugs of the Vater papilla associated with reflux of pancreatic juice into the bile duct cannot be excluded as an explanation for the rupture.

## 4. Conclusion

Recurrent pancreatitis in children justifies radiological visualisation of the biliary tree. MRCP enables to diagnose rare complications, such as a choledochal rupture. ERCP offers the opportunity for noninvasive treatment, also in children.

## Abbreviations

CBD: Common bile duct
MRCP: Magnetic resonance cholecystopancreaticography
ERCP: Endoscopic retrograde cholecystopancreaticography.
